# Decline in Vaccine-Type *Streptococcus pneumoniae* Serotypes Following Pneumococcal Conjugate Vaccine Introduction in Madagascar

**DOI:** 10.1093/infdis/jiab226

**Published:** 2021-09-01

**Authors:** Julia L Raboba, Vonintsoa L Rahajamanana, Emilson P R Andriatahirintsoa, Ainamalala C Razafindrakoto, Andry M Andrianarivelo, Marcellin Nimpa Mengouo, Yolande Vuo Masembe, Goitom G Weldegebriel, Linda de Gouveia, Jason M Mwenda, Annick L Robinson

**Affiliations:** 1 Department of Child Health, Teaching Hospital, Centre Hospitalier Universitaire Mère Enfant Tsaralàlana, Antananarivo, Madagascar; 2 Teaching Hospital, Centre Hospitalier Universitaire Anosiala, Antananarivo, Madagascar; 3 Teaching Hospital, Centre Hospitalier Universitaire Joseph Ravoahangy Andrianavalona, Antananarivo, Madagascar; 4 World Health Organization Country Office, Antananarivo, Madagascar; 5 World Health Organization Inter-Country Support Team East and Southern Africa, Harare, Zimbabwe; 6 Regional Reference Laboratory, National Institute of Communicable Diseases, Centre for Respiratory Diseases and Meningitis, Johannesburg, South Africa; 7 World Health Organization Regional Office for Africa, Brazzaville, Congo

**Keywords:** vaccine, *Streptococcus pneumonia*, serotype, surveillance

## Abstract

**Background:**

The 10-valent conjugate vaccine (PCV10) was introduced into the Extended Program on Immunization in Madagascar. We assessed the impact of PCV10 on the targeted pneumococcal serotypes among children < 5 years of age at Centre Hospitalier Universitaire Mère Enfant Tsaralalàna.

**Method:**

Between 2012 and December 2018, cerebrospinal fluid (CSF) samples were collected and tested for S. pneumoniae by culture, and antigen tests. The Sentinel Site Laboratory (SSL) referred available CSF samples to the Regional Reference Laboratory (RRL) for real-time polymerase chain reaction confirmatory testing and serotyping.

**Results:**

In total, 3616 CSF specimens were collected. The SSL referred 2716 to the RRL; 125 were positive for S. pneumoniae. At the RRL, 115 samples that tested positive for S. pneumoniae were serotyped; PCV10 serotypes accounted for 20%. Compared to the pre-PCV period, the proportion of S. pneumoniae detected declined from 22% to 6.6%, (P < .05), the proportion of PCV10 serotypes as the cause of pneumococcal meningitis cases declined by 26% following vaccine introduction.

**Conclusions:**

In our findings, PCV10 introduction resulted in a decline of meningitis caused by S. pneumoniae and PCV10 vaccine serotypes.

Streptococcus *pneumoniae* remains the most common cause of pediatric bacterial meningitis in the African region even after reported cases declined following pneumococcal conjugate vaccine (PCV) introduction [1]. Pneumococcal meningitis in children continues to be a serious disease with high fatality rate in developing and developed countries [2, 3], with a 14% of mortality rate in Madagascar [4]. Pediatric bacterial meningitis (PBM) surveillance in Madagascar for monitoring of *S. pneumoniae, Haemophilus influenzae,* and *Neisseria meningitidis* from lumbar puncture began in 2012. A recent study from Madagascar showed that about 80% of meningitis cases detected by the PBM network were caused by *S. pneumoniae*; children aged 1 month to < l year comprised the most cases (71%) [4].

In 2006, the World Health Organization (WHO) recommended the inclusion of PCV in all routine immunization programs, especially in countries with high pneumococcal disease burden [5]. The government of Madagascar included the H. *influenzae* type b (Hib) vaccine into the Extended Program on Immunization (EPI) in 2008 and the 10-valent PCV (PCV10) in October 2012. The meningococcal vaccine is as yet not available in the EPI program. A 3-dose infant vaccination of PCV10 without a booster is scheduled in the first 14 weeks of life (6, 10, and 14 weeks). PCV10 impact on pneumococcal meningitis and pneumonia hospitalizations in Madagascar was documented in a recent publication: bacterial meningitis fell from 4.5% to 2.6% of hospitalizations and pneumonia hospitalizations decreased from 24.5% to 19.0% [4]. However, the impact on the pneumococcus and vaccine-type serotypes in Madagascar is unknown. We assessed the impact of PCV10 on vaccine-type pneumococcal serotypes in children ≤5 years of age admitted for meningitis at the Centre Hospitalier Universitaire Mère Enfant Tsaralalàna (CHUMET). This is the first evaluation of the decline in *S. pneumoniae* and its serotypes following PCV10 and Hib vaccine introduction.

## METHODS

This study was conducted at CHUMET, the only sentinel PBM surveillance site in Madagascar. CHUMET reports to the WHO Global Invasive Bacterial Vaccine Preventable Diseases Surveillance Network. PBM surveillance was established in January 2012 at CHUMET. This hospital is an 82-bed public reference pediatric hospital located in the capital of Madagascar and primarily serves local patients, although some patients come from elsewhere in the country. Children ≤5 years old admitted to CHUMET who fulfilled the WHO case definition of suspected bacterial meningitis were eligible for enrollment. A lumbar puncture for routine diagnostic testing was performed on these children.

A suspected case of meningitis was defined as a child with sudden onset of fever (>38.5°C rectal or 38.0°C axillary) in combination with 1 of the following clinical signs: neck/head stiffness, altered consciousness with no other alternative diagnosis, or with other meningeal sign [6]. A probable case was a suspected case with cerebral spinal fluid (CSF) examination showing at least 1 of the following: turbid appearance, leukocytosis (>100 cells/mm^3^), leukocytosis (10–100 cells/mm^3^), and either an elevated protein (>100 mg/dL) or decreased glucose (< 40 mg/dL) [6].

Cases were deemed confirmed pneumococcal meningitis if a pneumococcus was detected in the CSF by 1 of the laboratory methods. CSF specimens collected from suspected cases were tested at the Sentinel Site Laboratory (SSL) and included chemical (glucose and protein concentrations) and microbiological analyses (Gram stain, culture, and rapid diagnostic assay) for the detection of *S. pneumoniae* antigen using Alere BinaxNOW Antigen Cards and/or Pastorex Meningitis Bio-Rad latex agglutination test. The Pastorex meningitis kit detects *H. influenzae* type b, *S. pneumoniae*, *N. meningitidis* group (A, B, C, W, and Y antigens), *Escherichia coli* K1, and group B streptococci.

*S. pneumoniae* isolates were cryopreserved in STGG (skimmed milk, tryptone, glucose, and glycerol). Any residual CSF was stored at −20°C and submitted together with available isolates to the Regional Reference Laboratory (RRL) at the National Institute for Communicable Diseases in South Africa, where real-time polymerase chain reaction (RT-PCR), was performed on all CSF specimens received. Total nucleic acid (DNA) was extracted using a MagNA pure 96 instrument (Roche) from each CSF received and extracts were run on an Applied Bio-systems 7500 Fast real-time PCR instrument (Applied Biosystems) for the molecular detection of *ctrA, lytA,* and *hpd* genes for confirmation of *N. meningitidis*, *S. pneumoniae,* and *H. influenzae*, respectively [7]. All *lytA*-positive CSFs underwent pneumococcal serotyping by performing 8 multiplex PCR reactions to identify 38 individual serotypes/serogroups [7, 8]. Ten of the 38 serotypes are included in PCV10 and the other 28 are non-PCV10 serotypes. Samples that were *lytA* positive but had a negative result in the serotyping PCR were grouped with non-PCV10 serotypes.

At the RRL, isolate identification was confirmed by standard laboratory methods and serotyped by the Quellung reaction using serotype-specific antisera (Statens Serum Institut). Minimum inhibitory concentration (MIC) to penicillin and ceftriaxone was performed by broth microdilution method using commercially available Sensititre-SASP2 panels (Trek Diagnostics). Isolates were nonsusceptible to penicillin if the MIC was ≥ 0.12 mg/L and to ceftriaxone if the MIC was >0.5 mg/L according to the Clinical and Laboratory Standards Institute breakpoints [9].

For each eligible case, a case report form was completed. Patient information collected included identity (name, date of birth, sex, and address), clinical information (diagnosis and date of admission, date of onset, prior antibiotic use, and clinical signs), vaccination status, outcome, and discharge (date and diagnosis). Information about vaccination status was confirmed by vaccine card; however, availability of vaccine card documentation was limited. Clinical, SSL data, and RRL results were captured in a database.

Cases reported in 2012 (9 months before and 2 months immediately following vaccine introduction) were considered pre-PCV immunization, and the period following vaccine introduction (January 2013 to December 2018) as post-PCV. χ ^2^ was calculated for testing the difference in positivity between the pre- and post-PCV time periods. *P* values <.05 were considered significant.

*S. pneumoniae* cases pre- and post-PCV were reported, including their serotype distribution by time periods, age groups, PCV10 serotypes (1, 4, 5, 6B, 7F, 9V, 14, 18C, 19F, and 23F), and non-PCV10 serotypes (serotypes other than these 10 PCV10 serotypes). The samples with mixed PCV10 and non-PCV10 types were considered to be non-PCV10 serotype.

This activity was considered routine public health surveillance by Ministry of Health and WHO. All analyses were performed using Microsoft Excel and R version 3.61.

## RESULTS

**Figure 1.**
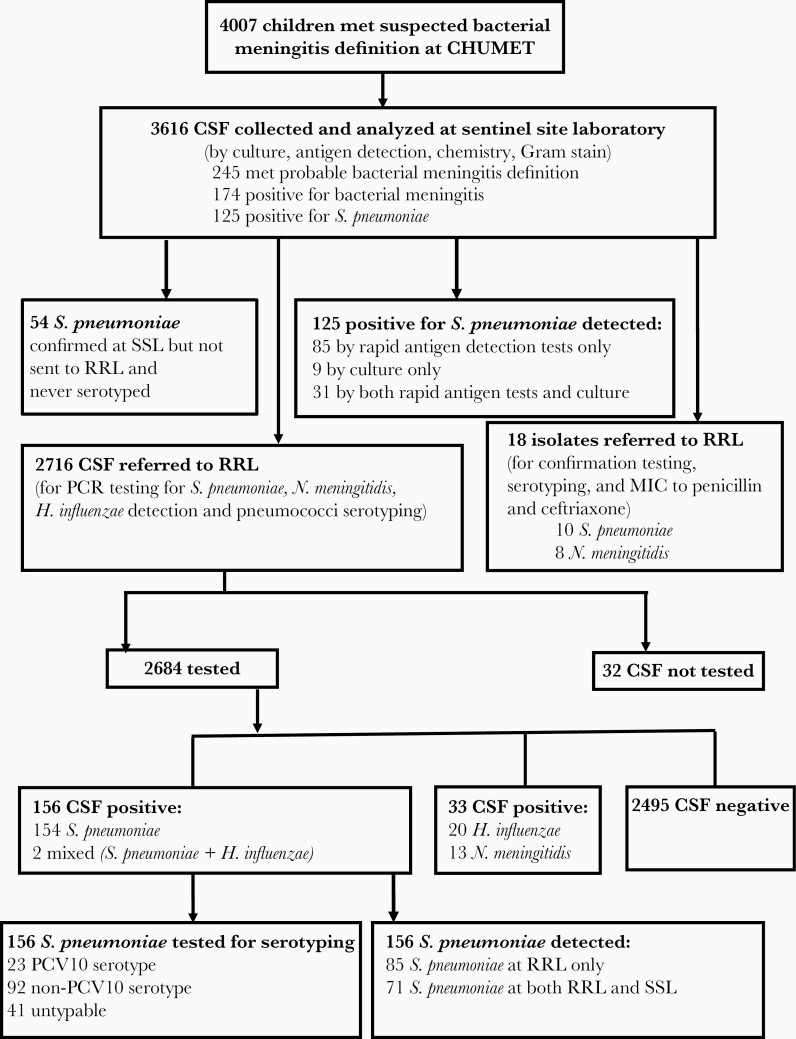
Study profile during the observation period 1 January 2012 to 31 December 2018. Testing was for detection of *Streptococcus pneumoniae, Neisseria meningitidis,* and *Haemophilus influenzae.* Abbreviations: CHUMET, Centre Hospitalier Universitaire Mère Enfant Tsaralalàna; CSF, cerebrospinal fluid; MIC, minimum inhibitory concentration; PCR, polymerase chain reaction; RRL, Regional Reference Laboratory; SSL, Sentinel Site Laboratory.

In total, 4007 children met the case definition for suspected bacterial meningitis; for 3616 (90.2%) cases CSF specimens were collected and analyzed by routine diagnostic tests at SSL. Of these, 245 were classified as probable bacterial meningitis and 174 were confirmed bacterial meningitis: 72% (n = 125) due to *S. pneumoniae,* 1% (n = 1) *H. influenzae,* 9% (n = 16) *N. meningitidis*, 9% (n = 15) group B streptococci, 3% (n = 6) *E. coli*, and 6% (n = 11) other bacteria of doubtful significance (Table 1). Of these bacteria detected, 110 were confirmed by rapid antigen detection tests only, 29 by culture only, and 35 were detected by both culture and rapid antigen detection tests (Table 1). Of the *S. pneumoniae* confirmed cases, 85 were detected by rapid antigen detection tests only (by Alere BinaxNOW Antigen Cards, and/or Pastorex Meningitis Bio-Rad latex agglutination test), 9 by culture only, and 31 were by both culture and rapid antigen detection tests (Table 1). Of the 3616 collected specimens, 75% of CSF (n = 2716) were tested by PCR at RRL, of which 7% (n = 191) were positive for *S. pneumoniae, H. influenzae*, or *N. meningitidis*. Of those, *S. pneumoniae* was detected in 82% (n = 156), *N. meningitidis* was detected in 6.8% (n = 13), and *H. influenzae* was detected in 12% (n = 22) of CSF specimens. Over half (54%, n = 85) of the specimens where *S. pneumoniae* was detected by PCR at RRL were negative for *S. pneumoniae* by culture or antigen testing at SSL; 46% (n = 71) were positive by both PCR at RRL and routine diagnostic tests at SSL (Figure 1).

**Table 1.** Bacteria Detected at the Sentinel Site Laboratory Between 2012 and 2018, Stratified by Bacterial Tests of Confirmation

Bacteria Detected	Antigen Tests^a^ Only	Culture Only	Antigen Tests^a^ and Culture	Total
*Streptococcus pneumoniae*	85	9	31	125
*Haemophilus influenzae*	0	1	0	1
*Neisseria meningitidis*	6	7	3	16
Group B streptococci	15	0	0	15
*Escherichia coli*	4	1	1	6
*Haemophilus parainfluenzae*	0	2	0	2
*Klebsiella pneumoniae*	0	3	0	3
*Staphylococcus aureus*	0	2	0	2
*Bacillus cereus*	0	1	0	1
*Providencia alcalifaciens*	0	1	0	1
*Moraxella* sp	0	1	0	1
*Pantoea* sp	0	1	0	1
Total	110	29	35	174

^a^Antigen tests = rapid antigen detection tests.

**Figure 2.**
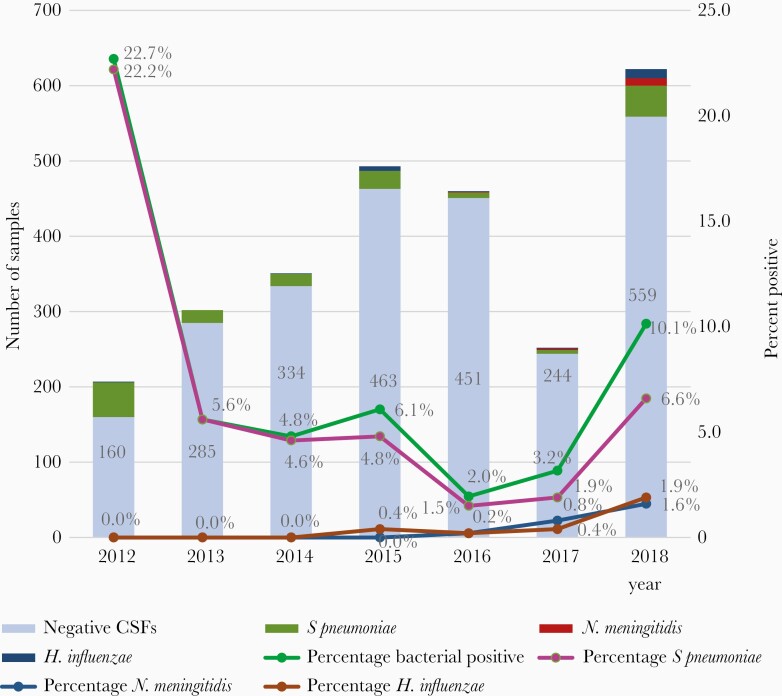
Number of positive and negative CSFs and percentage positive for bacteria and *Streptococcus pneumoniae, Neisseria meningitidis,* Haemophilus *influenzae* detected by PCR by year, 2012–2018. Abbreviations: CSF, cerebrospinal fluid; PCR, polymerase chain reaction.

Bacterial meningitis detected by PCR declined after PCV introduction. Of the 207 CSF collected in 2012 and tested at RRL, 23% (n = 47) were positive for bacterial meningitis, with *S. pneumoniae* detected in 22% (n = 46). During the first 5 full years after vaccination introduction, overall bacterial meningitis and meningitis due to *S. pneumoniae* decreased. Compared to the pre-PCV period, the proportion of suspected meningitis cases that were determined to be positive for *S. pneumoniae, H. influenzae,* or *N. meningitidis* fell from 23% to 10% following PCV introduction (2013–2018), and the proportion due to *S. pneumoniae* declined from 22% to 6.6% of suspected meningitis cases (*P* < .05). This represents a relative decrease of 57% in bacterial meningitis detected (*P* < .01) and 70% in *S. pneumoniae* detected (*P* < .01) compared to 2012. Throughout the study period (2012–2018), *S. pneumoniae* remained the most common pathogen detected compared with *H. influenzae* and *N. meningitidis*; however, the proportion of bacterial meningitis cases due to *S. pneumoniae* also decreased, from 97.9% to 76.4%. Year on year numbers of CSF specimens tested and the proportions determined to be bacterial, and those confirmed to be due to *S. pneumoniae*, *H. influenzae*, and *N. meningitidis,* are shown in Figure 2.

**Figure 3.**
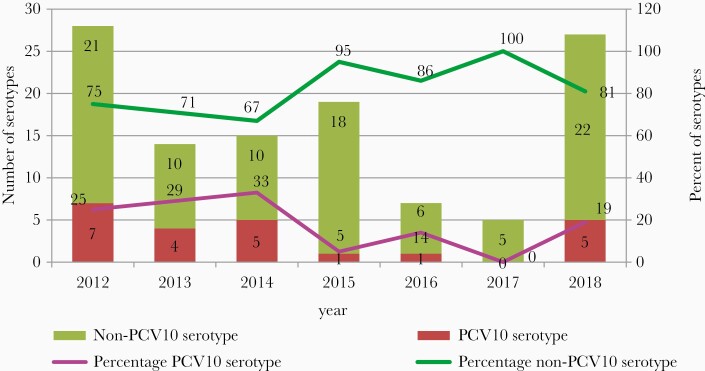
Number and percent of 10-valent pneumococcal conjugate vaccine (PCV10) and non-PCV10 *Streptococcus pneumoniae* serotypes detected by the Regional Reference Laboratory, by year 2012–2018.

Of the 156 *S. pneumoniae* detected at RRL, 74% (n = 115) were serotyped and for 26% (n = 41) a serotype determination was not possible due to low DNA concentration (high cycle threshold) in the samples. PCV10 serotypes accounted for 20% (23/115) of specimens collected during the 7 years of surveillance. The non-PCV10 serotypes included single non-PCV10 (2, 8,19A, 35B) and mixed serotypes (6A/6B, 12F/12A/12B/44/46, and 18A/18B/18C/18F) were 80% (92/115). The proportion of PCV10 serotypes as the cause of pneumococcal meningitis cases declined by 26% following vaccine introduction: 25% (7/28 in 2012) versus 18.4% (16/87, average among all cases reported in 2013–2018) (Figure 3). There was no difference between the mortality rate by PCV10 serotypes before and after vaccination introduction (before, 25%, n = 2; after, 25%, n = 4; Table 2). The decline in non-PCV10 serotypes is not statistically significantly different between pre-PCV and post-PCV (*P* = .41). All *S. pneumoniae* isolates (n = 10) were susceptible to both penicillin (MIC ≤ 0.06 µg/mL) and ceftriaxone (MIC ≤ 0.5 µg/mL).

**Table 2.** Characteristics of Pneumococcal Meningitis Between 2012 and 2018, Stratified by PCV Serotypes

Characteristics	Suspected Cases (n = 4007)		All *Pneumococcus* Detected (n = 156)		PCV10 Serotype (n = 24)		Non-PCV10 Serotype		*P* Value^a^
	Pre-PCV (n = 607)	Post-PCV (n = 3400)	Pre-PCV (n = 46)	Post-PCV (n = 110)	Pre-PCV (n = 8)	Post-PCV (n = 16)	Pre-PCV (n = 20)	Post-PCV (n = 71)	
Age groups									
Neonate	71 (11.7)	411 (12.1)	0 (0)	7 (6.4)	0 (0)	1 (6.2)	0 (0)	4 (5.6)	…
1 mo to <1 y	294 (48.4)	1306 (38.4)	35 (76.1)	75 (68.2)	7 (87.5)	9 (56.2)	17 (85)	57 (80.3)	…
1 to <3 y	199 (32.8)	1335 (39.3)	10 (21.7)	24 (21.8)	1 (12.5)	5 (31.3)	2 (10)	10 (14.1)	…
3 to <5 y	43 (7.1)	348 (10.2)	1 (2.2)	4 (3.6)	0 (0)	1 (6.2)	1 (5)	0 (0)	…
Sex									
Male	350 (57.7)	1921 (56.5)	26 (56.5)	65 (59.1)	3 (37.5)	12 (75)	13 (65)	40 (56.3)	…
Female	25742.3)	1479 (43.5)	20 (43.5)	45 (40.9)	5 (62.5)	4 (25)	7 (35)	31 (43.7)	…
Mortality	3 (0.49)	225 (6.6)	6 (13)	14 (12.7)	2 (25)	4 (25)	4 (20)	9 (12.7)	…
CSF characteristics									
Turbid/cloudy	21 (3.5)	105 (3.1)	11 (23.9)	50 (45.5)	3 (37.5)	8 (50)	7 (35)	36 (50.7)	>.05
White cells count, >100 cells/mm^3^	25 (4.1)	102 (3)	6 (13)	34 (30.9)	2 (25)	5 (31.3)	3 (15)	33 (46.5)	>.05
Protein >100 mg/dL	35 (5.8)	312 (9.2)	10 (21.7)	49 (44.5)	3 (37.5)	5 (31.3)	6 (30)	40 (56.3)	>.05

Data are No. (%).

Abbreviation: CSF, cerebrospinal fluid; PCV, pneumococcal conjugate vaccine.

^a^*P* value: PCV10 serotype and non-PCV10 serotype.

**Figure 4.**
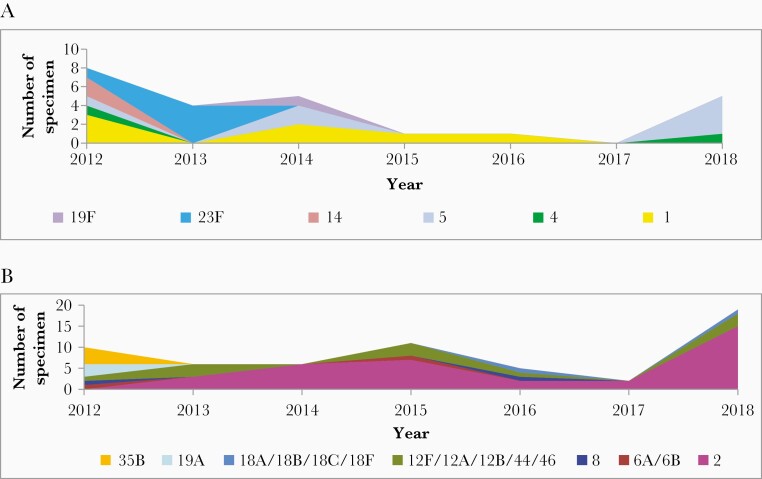
Annual distribution of *Streptococcus pneumoniae* serotypes, 2012–2018; (*A*) PCV10 serotypes and (*B*) non-PCV10 serotypes.

Overall, the predominant PCV10 serotypes (serotypes 1 and 23F) declined during the period after PCV introduction: serotype 1 was the most commonly detected serotype in 2012, declined in 2013–2016, and was not detected in 2017–2018; serotype 23F was the most commonly detected PCV10 serotype in 2013 and was not detected in 2014–2018. However, the percentage of *S. pneumoniae* due to serotype 2, a non-PCV10 serotype, increased after vaccination introduction and became the most commonly circulating serotype (Figure 4).

Nearly all of the pneumococcal meningitis cases detected were children aged 1 month to <1 year and children in the 1 to <3-year-old age group: 76.1% of pneumococcal meningitis cases (pre-PCV introduction) and 68.2% (after PCV) occurred in the 1 month to <1-year-old age group, while 21.7% and 21.8% of pneumococcal meningitis cases occurred in the 1 year to <3-year-old age group pre- and post-PCV introduction, respectively. Both PCV10 and non-PCV10 *S. pneumoniae* serotypes disproportionately affected these age groups. The proportion of PCV10 serotypes detected among children aged 1 month to <1 year decreased significantly from 87.5% (n = 7) before vaccination introduction to 56.2% (n = 9) after (*P* = .01); 1 month to <1 year olds represented a similar proportion of non-PCV10 serotypes before PCV10 vaccine introduction (85%, n = 17) but had less decline after (80%, n = 57) (Table 2). Pneumococcal meningitis was rare in neonates during both time periods (0% before and 6.4% after vaccination introduction; Table 2). There was no substantial difference in the characteristics of CSF specimens of pneumococcal meningitis cases due to PCV serotypes compared to non-PCV serotypes in this study (*P* > .05; Table 2).

## Discussion

This is the first evaluation on the change of *S. pneumoniae* serotypes after PCV10 introduction in Madagascar. Vaccination information was rarely available from participating children thus the percentage of children enrolled in this study who received PCV10 vaccine is unknown in this population. However, Madagascar national coverage for full vaccination with PVC10 has been not high (69%–76% in 2013 to 2018) [10].

Except among neonates, who are too young to receive PCV10 vaccine, and in 2018 when pneumococcal meningitis increased, the incidence of PCV10 serotypes decreased following PCV10 vaccine introduction. The decline was not only observed in pneumococcal meningitis with PCV10 serotypes but also in bacterial meningitis, pneumonia, and total hospitalizations. The decrease in total hospitalizations could be due to the impact of Rotarix, introduced in May 2014, on diarrhea as well as the impact of PCV10 [11]. Also, during the evaluation period, the country has been implementing various child survival interventions during the annual African vaccination week (like deworming and vitamin A supplementations, breast feeding, and hand washing) [12, 13].

In 2018, the increase of incidence of bacterial meningitis, and confirmed for *H. influenzae, N. meningitidis,* and *S. pneumoniae,* may have been due to the increase of number of enrolled cases. As the number of tested CSF samples increased so did the chance to detect more bacteria. The increase of PCV10 serotypes was a result of the reappearance of serotype 5.

The incidence of non-PCV10 serotypes increased in 2015–2018; this may have been related to serotype replacement phenomenon by serotype 2 (56% in 2018). Also, serotype 2 was an issue with meningitis in Bangladesh where it emerged before PCV10 introduction [14]. The replacement phenomenon has been observed in other settings, but serotype 2 has not commonly demonstrated serotype replacement [15, 16]. The decline of PCV10 serotypes was similar to the findings in some African and European countries that introduced the PCV vaccine. In Burkina Faso, the overall annual incidence of meningitis due to PCV13 serotypes decreased each year since the introduction of PCV13 by 62% [17]; similarly, after PCV introduction, there were reductions in the rates of disease caused by PCV7 in South Africa [18]; PCV7 serotypes have almost disappeared in children in European countries with high vaccine uptake [15] and there has been a decrease on the incidence of vaccine-type invasive pneumococcal disease in all age groups in Norway [16].

Our study showed that, overall, the predominant PCV10 serotypes (serotypes 1 and 23F) declined during the period after PCV introduction. Similarly, in South Africa, these 3 serotypes were among the PCV serotypes to decrease after PCV implementation [19]. In Mozambique, serotypes 1 and 5 remained the most common serotype in the 1 year immediately after vaccination [20] and the effect of PCV13 against serotype 1 was not yet evident in Gambia [21].

All *S. pneumoniae* isolates tested at RRL showed susceptibility to both penicillin and ceftriaxone. Despite the low number of isolates tested, this finding is similar to other studies showing the rate of antibiotic-resistant invasive pneumococcal infections decreased in young children after the introduction of the conjugate vaccine [22]. The study on the serotype of *S. pneumoniae* isolates was not done because the serotype of each isolate was the same as that detected in the original CSF.

The limitations to our study include that it was conducted in only 1 pediatric hospital in Madagascar and may not be representative of trends at other facilities. However, children from outside of the capital city do receive care at this sentinel facility. Additionally, the real percentage of vaccine coverage for the children enrolled in this evaluation was unknown because of the large number of children who had no documented vaccination cards. Finally, the study period before PCV10 introduction was less than 1 year and the overall number of *S. pneumoniae* isolates was low. However, the number of *S. pneumoniae* that were not serotyped was also low.

## CONCLUSION

Our study highlights the impact of PCV10 introduction into the national immunization program in Madagascar by reducing pneumococcal meningitis in children aged < 5 years as well as a decline in vaccine-type *S. pneumoniae* serotypes. Ongoing surveillance is essential to keep monitoring these changes, including serotype replacement, to guide further policy decisions [23].

## Notes

**Acknowledgments.** We thank the medical staff, medical trainee, and staff at the laboratory from Centre Hospitalier Universitaire Mère Enfant Tsaralàlana, Antananarivo; the World Health Organization; the Direction de Programme Elargi de Vaccinations, Madagascar; and laboratory staff at the National Institute for Communicable Disease of the National Health Laboratory Service, Johannesburg, South Africa.

***Supplement sponsorship.*** This supplement is sponsored by the World Health Organization and the U. S. Centers for Disease Control and Prevention.

**Potential conflicts of interests.** All authors: No reported conflicts of interest. All authors have submitted the ICMJE Form for Disclosure of Potential Conflicts of Interest. Conflicts that the editors consider relevant to the content of the manuscript have been disclosed.
